# A novel periodontal endoscopy-aided non-incisional periodontal regeneration technique in the treatment of intrabony defects: a retrospective cohort study

**DOI:** 10.1186/s12903-023-03674-9

**Published:** 2023-12-03

**Authors:** Jiahong Shi, Jinmeng Wang, Zhiyu Yang, Jingwen Li, Lang Lei, Houxuan Li

**Affiliations:** 1 Department of Periodontics, Nanjing Stomatological Hospital, Affiliated Hospital of medical School, Nanjing University, Nanjing, China; 2 Department of Orthodontics, Nanjing Stomatological Hospital, Affiliated Hospital of medical School, Nanjing University, Nanjing, China

**Keywords:** Intrabony defects, Periodontal regeneration, Periodontal endoscopy, Periodontitis

## Abstract

**Background:**

Gingival recession and post-operation discomfort are still a problem for patients receiving the periodontal regeneration surgery for intra-bony defects. To further reduce the trauma and the post-operation gingival recession, a novel periodontal endoscopy-aided non-incisional regeneration technique (NIT) was proposed in the treatment of intra-bony defects.

**Methods:**

Retrospective analysis of 21 subjects treated with NIT and 21 subjects with periodontal endoscopy-aided scaling and root planing (PSRP) at baseline and 1-year evaluation was conducted. After removing the subgingival calculus and granulation tissue, bone grafting materials were placed into intrabony defects with the assistance of a gingival retractor in the NIT group. Probing depth (PD), gingival recession (GR), clinical attachment level (CAL), as well as the distance between bone crest (BC) level and base of the defect (BD) (intrabony defect depth, IBD) were evaluated at baseline and 1 year after treatment.

**Results:**

At 1-year follow-up, the value of CAL, PD and IBD were statistically significant different compared with baseline in both two groups (*p*<0.001). CAL gain (*p* = 0.012) and PD reduction (*p =* 0.004) was greater in the NIT than PSRP. However, no difference in the IBD reduction was found between the NIT group and PSRP. Better CAL gain and PD reduction was achieved in the 1-year term in the NIT when compared with PSRP.

**Conclusion:**

NIT have resulted in significant gains in both clinical and radiographic parameters. NIT might be utilized as an alternative of the surgical treatment for periodontal intrabony defects.

**Trial registration:**

This clinical trial registration was registered retrospectively (August 3, 2023) and the number is ChiCTR2300074317.

## Introduction

Periodontitis, one of the most prevalent inflammatory diseases of humanity, is characterized by loss of tooth-supporting structures [1]. Consistent periodontal inflammation and alveolar bone destruction may lead to horizontal or vertical bony defects. Such deep bony defects post a great challenge for an adequate anatomical and visual access, which greatly limits the successful infection control [2]. Several approaches, such as scaling and root planing (SRP), open flap debridement, and periodontal regenerative surgical therapies, have been proposed for the repair of intrabony defects [3]. The periodontal regenerative therapies show great superiority over open flap surgery in the treatment of intrabony defects [4]. Despite great advances in the surgical procedures in the last two decades, gingival recession and post-operation discomfort is still a problem for patients as well as periodontists [4].

In the conventional regenerative surgery, access was acquired by flaps without preservation of the inter-dental tissues, which compromises primary wound closure and clot stability leading to a higher risk of gingival recession [5]. Later on, the minimally invasive surgery (MIST) that preserves interdental papilla was introduced, which greatly improves the would-healing process [6]. Several modifications of MIST have been made to further reduce trauma and improve treatment outcome. The single flap approach (SFA) was proposed to access the defect with one-side periodontal flap while preserving the opposite side [7]. The modified minimally invasive surgical technique (M-MIST), as a specific type of SFA technique, was aimed to preserve the entire gingiva by limiting the minimal incision at the buccal side [8]. The “entire papilla preservation (EPP)” technique was designed to access the defect from the neighbouring inter-dental space [9]. The modification of EPP technique, which is similar to tunnel-like approach, was intended to avoid incision at the sites of intrabony defects [10]. Satisfactory periodontal healing has been reported in the MIST-based surgeries [11–13]. From open flap surgery without preservation of the papilla, minimally invasive surgery (MIST), modified minimally invasive surgical technique (M-MIST) to entire papilla preservation (EPP), periodontists are in pursuit of clinical techniques with less invasion, better compliance and outcome.

By providing visual access and reduce surgical trauma, endoscopes have been widely used in treating sialolithiasis, orthognathic surgery and temporomandibular joint disorders in the dentistry in recent years [14]. In addition, the endoscopic system has been applied in the discipline of periodontics in the early 20th century [15]. The periodontal endoscopic system was composed of an imaging, lighting and magnification technology [16]. The periodontal endoscopic system allows direct visualization of subgingival biofilms, root surfaces, and dental calculus in periodontal pockets. The operator removes subgingival deposits based on real-time magnified images and the periodontal treatment may be less invasive [17, 18].

Previously, the periodontal endoscopic system is mainly used to remove calculus and biofilms in SRP procedures [16, 18–20]. Since periodontal endoscope may provide visual access to the intrabony wall, it may help the regeneration process by avoiding the elevation of flap. However, whether periodontal endoscope can be applied in cases with intrabony defects to aid periodontal regeneration has never been reported.

The formation is the first step of healing phase, and the stability of initial blood clot is the basis for optimal wound healing for bone regeneration [21]. The blood clot is rich in growth factors, cytokines and signaling molecules. After remodeling into highly vascularized granulation tissues, blood clots serve as natural scaffolds for bone formation [22, 23]. Based on the hypothesis that the stability of blood clot can improve without open flap [3, 24], this study was the first to propose the periodontal endoscopy-aided non-incisional regeneration technique (NIT) for the treatment of intrabony defects. In NIT, full access to the defect was acquired by the periodontal endoscopy rather than elevation of flaps, while bone substitutes were placed in the debrided defect with the aid of periodontal endoscopy. NIT method was brought forward to further reduce the invasion of periodontal regeneration surgery.

This study is based on the hypothesis that non-incisional procedure and the adjunctive use of bone graft materials may improve the results of periodontal regeneration. Thus, the aim of this study was to explore the feasibility of NIT for the repair of deep intrabony defects, and to compare its efficacy with periodontal endoscopy-aided SRP (PSRP).

## Method and materials

### Experimental design and ethical aspects

This study was a retrospective analysis of subjects who were referred to the Department of Periodontics, Nanjing Stomatological Hospital, Affiliated Hospital of medical School, Nanjing University between October 2018 and December 2021. This retrospective study involving human participants was in accordance with the ethical standards of the institutional and national research committee and with the 1964 Helsinki Declaration and its later amendments or comparable ethical standards. Institutional Review Board of Nanjing Stomatological Hospital approved this study (No. NJSH-2023NL-002). This trial was registered at clinical trial registry (Registration number: ChiCTR2300074317).

### Study population

Data were collected from treatment records of cases receiving periodontal endoscopy-aided treatment in Department of Periodontics, Nanjing Stomatological Hospital, Affiliated Hospital of Medical School, Nanjing University. The subjects were recruited between October 2018 and December 2021, and all the subjects had a follow-up of one year in January 2023.

All the subjects were treated according the EFP guidelines [25]. The subjects received phase I-II periodontal treatment, including oral hygiene instructions, supragingival dental cleaning and conventional scaling and root planing (SRP). Subjects were re-examined for periodontal clinical measurements at 6 weeks after periodontal cause-related treatment. Surgical treatment was recommended for subjects with periodontal pockets ≥ 5 mm with bleeding on probing after step 2 of periodontal treatment performed. To control potential sources of bias, all of the subjects were well informed of the potential benefit and risk of NIT or PSRP treatment. The allocation of subjects was mainly based on the cost, surgery time and patients’ acceptance of new technique.

**Fig. 1:**
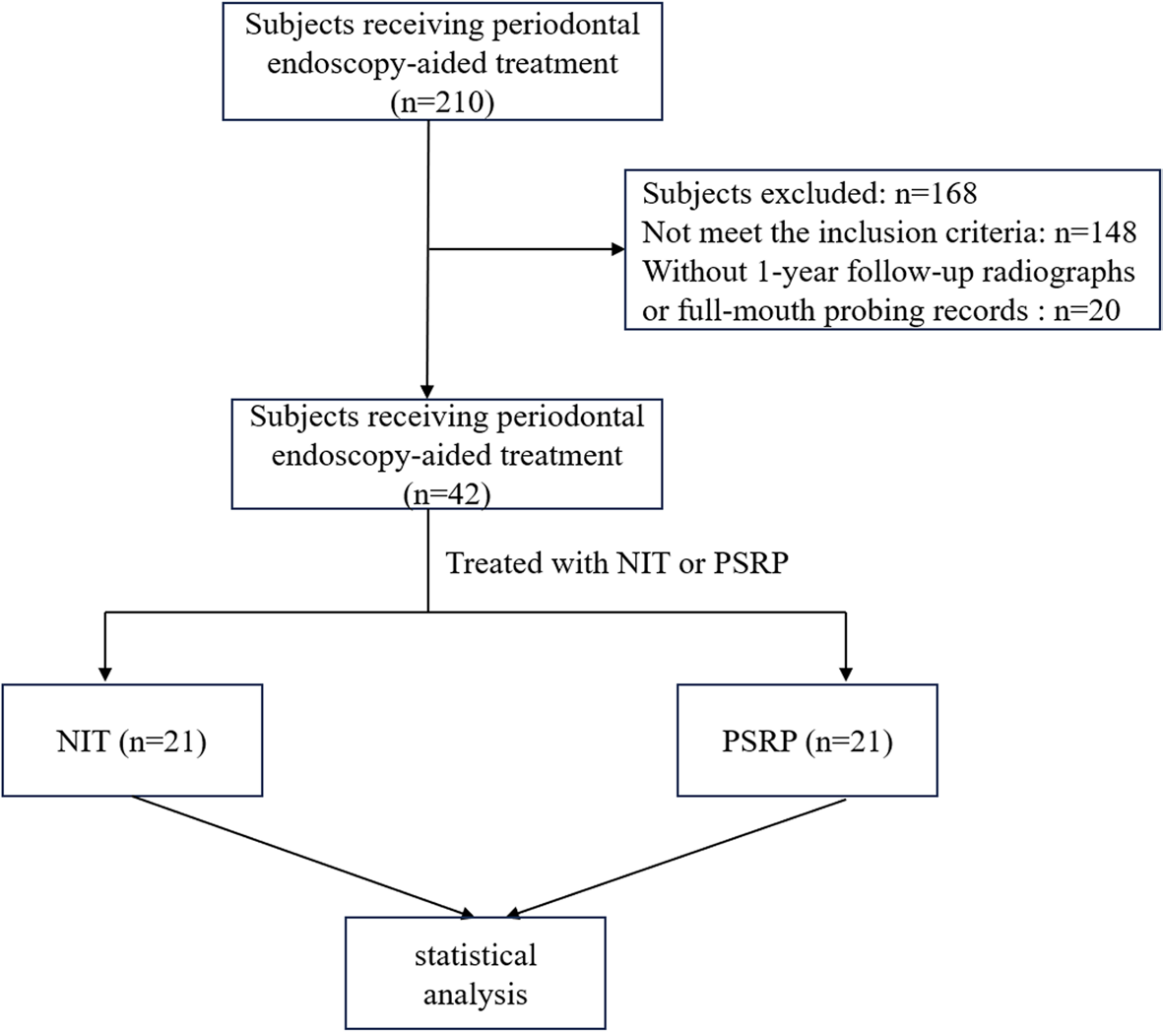
The flowchart of study design. NIT, periodontal endoscopy-aided non-incisional regeneration technique; PSRP, periodontal endoscopy-aided scaling and root planing

The subjects were included in this study when the inclusion criteria were satisfied: (1) diagnosed with stage III/IV periodontitis according to 2018 new classification of periodontal and peri-implant diseases and conditions [26]; (2) without systematic diseases; (3) without medications influencing periodontal condition in previous six months; (4) without history of smoking; (5) not pregnant or lactating; (6) full mouth plaque score (FMPS) ≤ 20% and full mouth bleeding score (FMBS) ) ≤ 25%; (7) at least one tooth with probing depth (PD) ≥ 5 mm and bleeding on probing associated with an interproximal intrabony defect in radiographs treated by NIT or PSRP; (8) at least one intrabony defect with the depth ≥ 3 mm. Consequently, 42 subjects, contributing 117 intrabony sites, were included in this study. Clinical parameters were recorded at baseline (6 weeks after completion of cause-related treatment) and 1 year after NIT or PSRP. The flowchart of this study was showed in Fig. 1.

### Sample size calculation

The difference in clinical attachment gain with or without Bio-Oss Collagen was about 1.5 mm and the variation was about 1.4 mm [8, 27]. The number of participants was calculated with 0.05 alpha error and 0.8 power. As a minimum, 18 participants were needed for each group.

### Surgical procedure

**Fig. 2:**
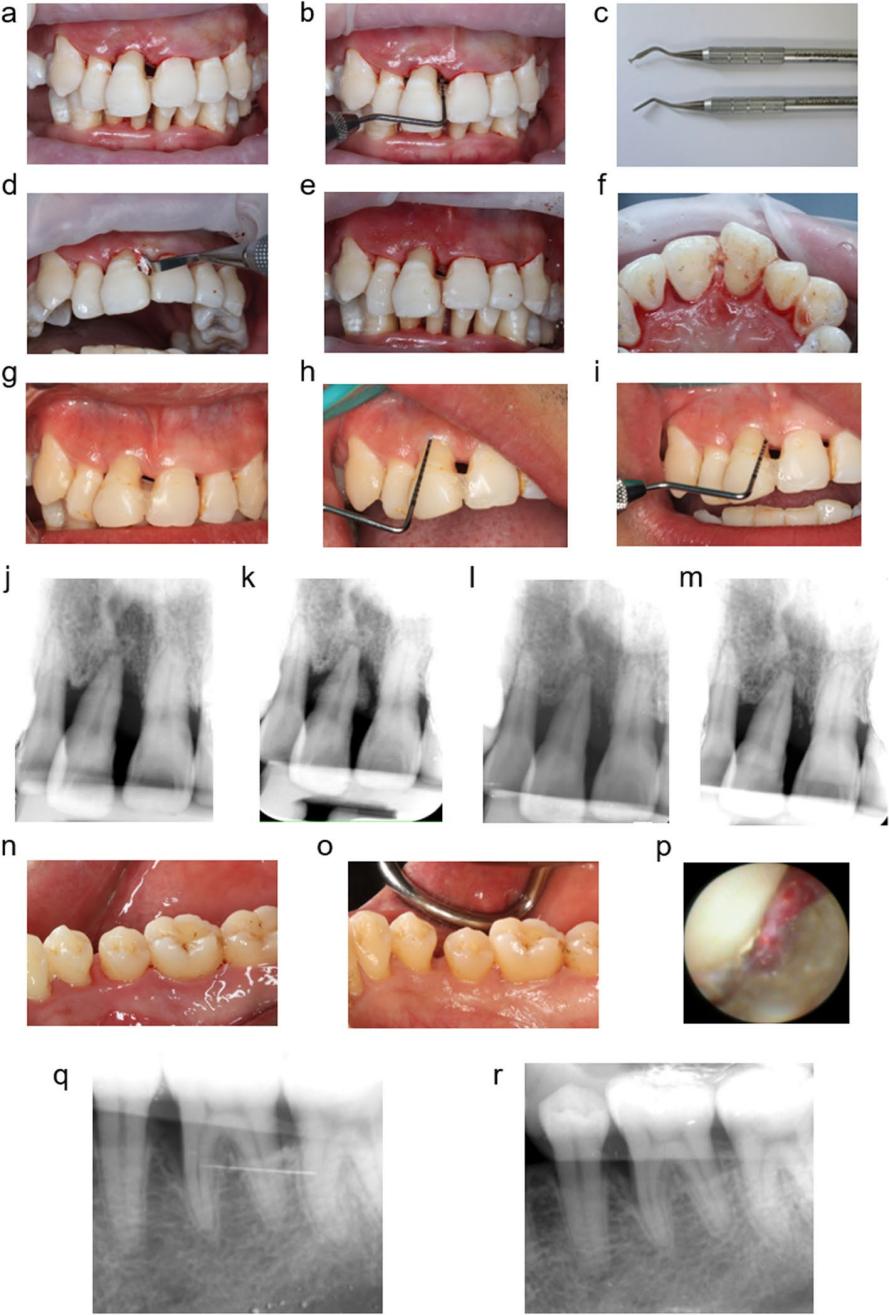
Typical cases treated by periodontal endoscopy-aided non-incisional periodontal regeneration technique (NIT) or periodontal endoscopy-aided scaling and root planing (PSRP). **a**-**m** a severe case treated with NIT. **a** Clinical image of buccal side of the right maxillary central incisors. **b** 10 mm probing depth (PD) was detected at buccal side. **c** Gingival retractor. **d** The placement of bone substitute (Bio-Oss collagen). **e**-**f** Clinical image of buccal and palatal side after the placement of bone substitute. **g**-**i** Clinical image of buccal side at 1-year evaluation after NIT. 2-4 mm PD was measured. **j** Baseline radiograph. **k** Periapical radiograph immediately after NIT. **l** Periapical radiograph at 3 months after the treatment. **m** Periapical radiograph at 1-year after the treatment. **n**-**r** a case treated with PSRP. **n** Clinical image of the left mandibular first molar at baseline. **o** Clinical image of the left mandibular first molar at 1-year after the treatment. **p** Subgingival field under the periodontal endoscope. **q** Baseline radiograph. **r** Periapical radiograph at 1-year after PSRP

All procedures were carried out by the same experienced clinician under a periodontal endoscope (Perioscopy Inc, Oakland, USA). Tooth with mobility > grade 1 according to Miller Index [28] needed to be stabilized with superbond C&B bonding system prior to periodontal treatment. Typical cases were showed in Fig. 2. The teeth involved in the experiment were treated with scaling and root planing by ultrasonic working tips (Piezon Master 700, Switzerland). Teeth were debrided under local infiltration anesthesia (Articaine-epinephrine) until no residual calculus could be detected under magnification of × 48 with the assistant of the periodontal endoscopy. In the NIT group, granulation tissue was removed with a mini curette (YOUNGER-GOOD, Hu-Friedy, USA). Bone filling materials, Bio-Oss collagen (Geistlich Biomaterials, Switzerland), was divided into small pieces. Bone grafting materials were soaked in sterile saline for 5 min. A delicate gingival retractor (SETO, Gum Protector, Japan) was used to retract the gingiva and small pieces of bone filling materials was placed into the bone defects with another gingival retractor repeatedly. The Bio-Oss collagen graft material was packed slightly overfilled. The gingival margin was gently compressed by sterile wetting gauzes for 1 min and no suture was used to fix. The periodontal dressing (Perio care, PULPDENT, USA) was placed to cover the gingival margin to maintain the stability of soft tissues and bone filling materials, which was removed after 7 days.

### Postsurgical care

Postoperative instructions including preventing flossing and chewing in the surgical site for 2 weeks. Subjects were instructed to rinse with 0.2% chlorhexidine twice daily for 2 weeks [29]. Professional tooth cleaning was performed every 3 months for 1 year. After the 1-year re-evaluation, supportive periodontal therapy (SPT) was performed at least every 6 months.

### Clinical and radiographic measurements

Clinical parameters including probing depth (PD), gingival recession (GR) and clinical attachment level (CAL) at the experiment sites were taken before the surgery (baseline) and 1-years after NIT or PSRP. All clinical examinations were performed by one examiner who was calibrated to reach reliability and consistency with a periodontal probe with 1 mm increments (UNC-15, Hu-Friedy, Chicago, USA). Repeated measurements of PD, CAL and GR were performed in 5 patients (not included in this study). The agreement of both clinical parameters within 1 mm between two repeated measurements (24 h apart) were > 0.98. PD was the distance between the gingival margin and the bottom of the periodontal pocket. GR was the distance between the cementoenamel junction (CEJ) and the gingival margin. CAL was calculated as the sum of PD and GR. The site with deepest PD at baseline between two mesial/ distal sites was selected for the following analysis. For FMPS, the presence of dental plaque on the buccal and lingual sides was recorded, and FMPS was equal to the percent of surfaces with plaque. Similarly, bleeding on probing was recorded at 6 sites of each tooth, and the FMBS was also calculated.

Digital periapical radiographs using the parallel technique were taken prior to and 1 year after NIT or PSRP treatment. The parameters of radiographs were set at 0.20 s exposure time and constant potential 7 mA 70 kV. The depth of the osseous defect was calculated on digital radiographs. The anatomical landmarks including the bone crest (BC) level and base of the defect (BD) were marked on the radiographs [30]. The distance between BC and BD were considered as the intrabony defect depth (IBD). IBD was measured with measurement tool in PACS/RIS system (Firtech Technology, China). The examiners who conducted the clinical and radiographic measurements were blinded to the allocation of subjects.

### Data analysis

Data were expressed as mean ± standard deviation (95% confidence interval) or number/percentage. The Shapiro-Wilk test was performed to test the normality. Comparisons between baseline and 1-year parameters were made using paired Wilcoxon test for parameters that were not normally distributed. Inter-group comparisons were made using Mann–Whitney U test for parameters that were not normally distributed. Comparisons between categorical variables were performed with Chi-square tests. All statistical analyses were performed with a statistics software (SPSS, IBM, version 25.0) and statistical comparisons were conducted at the 0.05 level of significance.

## Results

Forty-two subjects recruited from October 2018 until December 2021 were included in this study. The NIT group included 21 subjects (mean age 32.67 ± 5.83 years), contributing 55 intrabony sites. The PSRP group included 21 subjects (mean age 35.76 ± 9.63 years), contributing 62 intrabony sites. The characteristics of subjects and intrabony sites were showed in Table 1. No difference was detected in sex, age, tooth types, FMPS and FMBS between two groups. According to the value of PD, CAL and IBD, the baseline of the two experimental groups matched well (*p*>0.05) (Table 2). GR in the NIT group (0.55 ± 1.02 mm) was smaller than the PSRP group (0.96 ± 1.03 mm) (*p* = 0.006).

**Table 1:** Characteristics of study subjects at baseline and 1-year follow-up (n/n%/ mean ± SD)

Variables	NIT group	PSRP group	*p* value
Number of subjects	21	21	
Sex(males/females)	10/11	9/12	*p =* 1.0
age	32.67 ± 5.83	35.76 ± 9.63	*p =* 0.32
Systemic diseases	0	0	
Number of defects	55	62	
Tooth type			*p =* 0.195
molars	34 (62.5%)	38 (61.3%)	
premolars	5 (8.9%)	12 (19.4%)	
Incisors, canines	16 (28.6%)	12 (19.4)	
Tooth position			*p =* 0.025
Maxillary teeth	22 (39.3%)	13 (30.0%)	
Mandibular teeth	33 (60.7%)	49 (70.0%)	
FMPS (baseline)	12.2 ± 5.6	12.8 ± 5.4	*p =* 0.764
FMPS (1-year)	12.2 ± 5.0	11.8 ± 4.6	*p =* 0.856
FMBS (baseline)	10.0 ± 2.1	9.8 ± 2.2	*p =* 0.835
FMBS (1-year)	10.2 ± 2.1	9.8 ± 1.6	*p =* 0.408

*FMPS* full-mouth plaque score, *FMBS* full-mouth bleeding score

**Table 2:** Characteristics of intrabony defect sites at baseline (mean ± SD)

Variables	NIT group (*n* = 55)	PSRP group (*n* = 62)	
	baseline	baseline	*p* value
PD (mm)	7.55 ± 2.04	6.88 ± 1.93	*p =* 0.052
95%CI	(6.99, 8.10)	(6.36, 7.39)	
GR (mm)	0.55 ± 1.02	0.96 ± 1.03	*p =* 0.006
95%CI	(0.27, 0.81)	(0.69, 1.24)	
CAL (mm)	8.05 ± 2.30	7.84 ± 1.92	*p =* 0.447
95%CI	(7.47, 8.71)	(7.32, 8.35)	
IBD (mm)	4.69 ± 1.87	4.51 ± 1.55	*p* = 0.825
95%CI	(4.18, 5.19)	(4.09, 4.92)	

Comparison between baseline in the NIT group and the PSRP group

*PD* probing depth, *GR* gingival recession, *CAL* clinical attachment level, *IBD* radiographic intrabony defect depth

### Evaluation of post-surgery period

Apart from mild swelling and minor pain, all subjects in both groups complained of minimal discomfort after the surgery. No adverse postoperative complications, such as abscess, suppuration, edema or exposure of bone graft materials, were found. Only one patient reported very limited pain for the first 24 h after the surgery in the NIT and took 300 mg ibuprofen to relieve the discomfort.

### Clinical and radiographic outcomes

After 1 year, the intrabony sites in both groups showed significant PD reduction, CAL gain, IBD reduction and gingival recession. Changes in the PD, CAL, IBD and GR between baseline and 1-year measurement were statistically significant both in the NIT and the PSRP (*p* < 0.001). The average residual PD was 3.40 ± 0.99 mm and 3.80 ± 1.49 mm in the NIT and PSRP group, respectively (Table 3).

**Table 3:** Characteristics of intrabony defect sites at baseline and 1-year re-evaluation (mean ± SD)

Variables	NIT group (*n* = 55)			PSRP group (*n* = 62)		
	baseline	1-year	*p* ^*a*^ value	baseline	1-year	*p* ^*b*^ value
PD (mm)	7.55 ± 2.04	3.40 ± 0.99	*p*<0.001	6.88 ± 1.93	3.80 ± 1.49	*p*<0.001
95%CI	(6.99, 8.10)	(3.14, 3.67)		(6.36, 7.39)	(3.40, 4.20)	
GR (mm)	0.55 ± 1.02	1.25 ± 1.25	*p*<0.001	0.96 ± 1.03	1.66 ± 1.44	*p*<0.001
95%CI	(0.27, 0.81)	(0.92, 1.59)		(0.69, 1.24)	(1.27, 2.05)	
CAL (mm)	8.05 ± 2.30	4.46 ± 1.86	*p*<0.001	7.84 ± 1.92	5.46 ± 2.03	*p*<0.001
95%CI	(7.47, 8.71)	(3.96, 4.97)		(7.32, 8.35)	(4.92, 6.01)	
IBD (mm)	4.69 ± 1.87	2.97 ± 1.17	*p*<0.001	4.51 ± 1.55	2.83 ± 0.92	*p*<0.001
95%CI	(4.18, 5.19)	(2.65, 3.28)		(4.09, 4.92)	(2.47, 2.98)	

*PD* probing depth, *GR* gingival recession, *CAL* clinical attachment level, *IBD* radiographic intrabony defect depth

^a^ comparison between baseline and 1-year follow-up in the NIT group

^b^ comparison between baseline and 1-year follow-up in the PSRP group

From baseline to 1-year follow-up, the average PD reduction was 4.14 ± 2.16 mm (NIT group) and 3.07 ± 1.66 mm (PSRP group) with significantly more PD reduction in the NIT (*p =* 0.004). Improvement in the CAL gains was higher in the NIT than the PSRP (*p* = 0.012). No difference was found regarding the changes in the GR (*p* = 0.232). Regarding the radiographic intrabony defect depth, a slight but not statistically significant increase of intrabony bone filling was showed in the NIT compared with the PSRP (*p* = 0.448) (Table 4). There was no obvious crestal resorption or apposition radiographically after 1 year in both the NIT and the PSRP group.

**Table 4:** Changes in clinical and radiographic parameters over the 1-year period after receiving the NIT or PSRP treatment (mean ± SD)

Variables	NIT group (*n* = 55)	PSRP group (*n* = 62)	*p* value
ΔPD(mm)	-4.14 ± 2.16	-3.07 ± 1.66	*p =* 0.004
Estimate	(-4.72, -3.55)	(-3.52, -2.63)	
ΔGR (mm)	0.71 ± 0.90	0.70 ± 1.15	*p =* 0.232
Estimate	(0.47, 0.95)	(0.39, 1.01)	
ΔCAL (mm)	-3.62 ± 2.70	-2.38 ± 1.60	*p =* 0.012
Estimate	(-4.36, -2.90)	(-2.80, -1.95)	
ΔIBD(mm)	-1.72 ± 1.49	-1.68 ± 1.15	*p =* 0.448
Estimate	(-2.12, -1.32)	(-1.99, -1.37)	

*PD* probing depth, *GR* gingival recession, *CAL* clinical attachment level, *IBD* radiographic intrabony defect depth

Eighteen out of the 55 defects (32.7%) reached CAL improvement ≥ 5 mm and 52.7% (29 out of 55 sites) showed PD reduction ≥ 4 mm in the NIT group. However, only 5 out of the 62 defects (8.1%) reached CAL improvement ≥ 5 mm and 29.0% (18 out of 62 sites) showed PD reduction ≥ 4 mm in the PSRP group (Table 5). p value of Chi-square tests was both 0.011 for CAL improvement and PD reduction.

**Table 5:** Frequency (n) and frequency distribution (%) of PD and CAL changes at 1-year re-evaluation

	CAL changes		PD reduction	
Clinical parameters (mm)	NIT (*n* = 55)	PSRP (*n* = 62)	NIT (*n* = 55)	PSRP (*n* = 62)
≤ 2	22 (40.0%)	35 (56.5%)	16(29.1%)	26 (41.9%)
3	10 (18.2%)	16 (25.8%)	10 (18.2%)	18 (29.0%)
4	5 (9.1%)	6 (9.7%)	6 (10.9%)	10 (16.1%)
5	7 (12.7%)	2(3.2%)	6(10.9%)	3(4.8%)
≥ 6	11 (20.0%)	3 (4.8%)	17 (30.9%)	5 (8.1%)

*CAL* clinical attachment level, *PD* probing depth

## Discussion

The rationale of the NIT is minimally invasive to reduce injury in the traditional flap surgery, and it is a periodontal surgical method to accomplish periodontal regeneration under the periodontal endoscopy. In this study, the 1-year follow-up results demonstrated the effectiveness of NIT in the treatment of intrabony defects. The pocket closure and gains of CAL in the NIT group were superior to the results in the PSRP group. Moreover, the CAL gains of 3.62 ± 2.70 mm in NIT was consistent with other clinical trials investigating the efficiency of periodontal regenerative therapy that included guided tissue regeneration (GTR) or enamel matrix derivative (EMD) [24]. Access flap surgery is a common practice to tackle with residual pockets after cause-related therapy, such as prophylaxis and SRP [5]. In contrast to the previously-reported flap elevation techniques, in the NIT adequate anatomical access is achieved by use of a delicate gingival retractor, while sufficient visual access is finished by application of periodontal endoscope system. The flapless procedure in the NIT further maintains sufficient blood supply by avoiding incision of interdental papilla. NIT may become an optional technique for intra-bony defects.

However, the decrease of IBD was considered no statistically significant between two groups. Reduction of periodontal pocket depth reached stable 6 months after surgery, but the gradual recovery of radiographic bone height continued for almost 1–3 years [31]. Therefore, longer follow-up is needed to find out the difference in the bone regeneration. In addition, radiographic examination may not be sensitive to observe the tissue regeneration. Furthermore, the number of bony walls and the morphology of defects at baseline was not compared in the two groups.

Videoscope-assisted minimally invasive periodontal surgery (VMIS) was proposed in 2017 and the long-term outcomes of VMIS revealed the superiority over traditional periodontal regenerative approach as well as minimally invasive regenerative approaches [32, 33]. However, such VMIS still needs a minimal flap elevation. A complete “gingival cavity” and a completely preserved interdental papilla in the bone defect ensure the best healing conditions [9]. Mario et al. demonstrated that the lack of properly designed instruments for retracting gingival tissues and poor visual acuity may affect the flapless regenerative approach particularly for the posterior area [34]. By application of a delicate gingival retractor, adequate space can be obtained to finish surgical placement of Bio-Oss collagen in our NIT.

Although direct real-time visualization of subgingival root surface, management of attached calculus and soft tissues are possible with the assistance of the periodontal endoscopy [18]; however, the incomplete removal of granulation tissues may be a problem for the regeneration procedure. Granulation tissues are composed of macrophages, leukocytes and fibroblasts, as well as disordered collagen fibers [35]. In the traditional flap surgery, the complete excision of granulation tissues could reduce local bleeding, improve local debridement, and make space for the graft materials. However, it is a rather controversial issue regarding to what extent granulation tissues should be excised or retained. In the tooth extraction socket, granulation tissues may be a source of progenitor cells or multipotent stem cells (MSCs). MSCs from granulation tissues can differentiate into autogenous bone and fills up the empty socket [35, 36]. Therefore, removal of granulation tissues during periodontal surgery may result in the loss of vital MSCs [37]. In site preservation surgery of severe periodontally-affected teeth, the intra-socket granulation tissue was preserved and reflected from the buccal side toward the palate, acting as a barrier membrane. Such preservation of granulation tissue facilitates primary flap closure especially sites with severe bone loss [38].

Despite a notable benefit in achieving periodontal regeneration by NIT, case selection is a critical issue in the generalization of the technique. The size of endoscopic instrument may limit the accessibility in several conditions, such as a small opening of the periodontal pocket, morphology in the furcation area and patients with small opening of the mouth. In addition, gingival stripping is one potential risk in patients with thin-scalloped periodontal biotype. Moreover, anomaly in the root anatomy, including the enamel pearl and palatal gingival groove may impede the application of NIT.

Nevertheless, this study was a retrospective cohort study and the limitations mainly comes from the nature of retrospective study. The lack of randomization may introduce the risk of selection bias, baseline was not matched well in terms of the GR and different results may attain with different allocation of patients. No conclusion could be drawn on the superior and interior of these two treatments. Therefore, this study may be regarded as a feasibility study which has great value for the follow-up randomized controlled trial. Despite favorable periodontal regeneration in the NIT, further studies are needed to prove its advantage in the periodontal regeneration therapy. First all, prospective and randomized clinical trials that compare the NIT with other traditional periodontal regeneration techniques are needed to be further explored. Secondly, additional clinical studies with larger sample size and longer observation time are also required. Thirdly, clinical and radiographic evaluation could not replace histopathological results and the absence of histopathological analysis of regenerated tissues is a significant limitation. The decrease of PD and CAL may come from the attachment of the long junctional epithelium [4]. Thus, the quality and type of healing after periodontal intervention required to be further identified. Moreover, for generalization of the NIT in the periodontal regeneration, more data from other population and various periodontal conditions should be further evaluated.

## Conclusions

Under the limitations of this study, the results confirmed that: (1) NIT may achieve more CAL gain and PD reduction compared with PSRP for the treatment of intrabony defects; (2) By avoiding flap elevation, NIT provides optimal periodontal microenvironment for periodontal regeneration and NIT may become an optional technique for intrabony defects.

## Data Availability

The datasets used and analyzed during the current study available from the corresponding author on reasonable request.

## Abbreviations

PD: Probing depth
GR: Gingival recession
CAL: Clinical attachment level
SRP: Scaling and root planing
MIST: Minimally invasive surgery
SFA: Single flap approach
EPP: Entire papilla preservation
CEJ: Cementoenamel junction
BC: Bone crest
BD: Base of the defect
IBD: Intrabony defect depth
NIT: Periodontal endoscopy-aided non-incisional regeneration technique
PSRP: Periodontal endoscopy-aided scaling and root planing
